# Harnessing modern web application technology to create intuitive and efficient data visualization and sharing tools

**DOI:** 10.3389/fninf.2014.00071

**Published:** 2014-08-26

**Authors:** Dylan Wood, Margaret King, Drew Landis, William Courtney, Runtang Wang, Ross Kelly, Jessica A. Turner, Vince D. Calhoun

**Affiliations:** ^1^The Mind Research Network and LBERIAlbuquerque, NM, USA; ^2^Department of Psychology, Georgia State UniversityAtlanta, GA, USA; ^3^Department of Electrical and Computer Engineering, University of New MexicoAlbuquerque, NM, USA

**Keywords:** open neuroscience, big data, neuroinformatics, data sharing, query builder, javascript

## Abstract

Neuroscientists increasingly need to work with *big data* in order to derive meaningful results in their field. Collecting, organizing and analyzing this data can be a major hurdle on the road to scientific discovery. This hurdle can be lowered using the same technologies that are currently revolutionizing the way that cultural and social media sites represent and share information with their users. Web application technologies and standards such as RESTful webservices, HTML5 and high-performance in-browser JavaScript engines are being utilized to vastly improve the way that the world accesses and shares information. The neuroscience community can also benefit tremendously from these technologies. We present here a web application that allows users to explore and request the complex datasets that need to be shared among the neuroimaging community. The COINS (Collaborative Informatics and Neuroimaging Suite) Data Exchange uses web application technologies to facilitate data sharing in three phases: Exploration, Request/Communication, and Download. This paper will focus on the first phase, and how intuitive exploration of large and complex datasets is achieved using a framework that centers around asynchronous client-server communication (AJAX) and also exposes a powerful API that can be utilized by other applications to explore available data. First opened to the neuroscience community in August 2012, the Data Exchange has already provided researchers with over 2500 GB of data.

## Introduction

Many of the questions faced by the human neuroimaging community can no longer be answered through studying small data sets due to the wide structural and functional variance between individual subjects. Instead, neuroimaging researchers need to look at large populations in order to accurately distinguish between overarching trends and individual outliers. Accumulating such large data sets can be time consuming and expensive—often prohibitively so. In response to this challenge, some members of the neuroimaging community are molding a new approach to data collection. This new approach has been dubbed Open Neuroscience, and it necessitates that individual researchers will openly share phenotypic, genotypic and neuroimaging data and collection methodologies (Milham, 2012).

Thus far, several large datasets and sharing platforms have been released in the spirit of the Open Neuroscience initiative with great support and success. One of the earliest examples was the fMRI Data Center (fMRIDC), which consolidated and shared thousands of datasets from 2000 to 2007 (Van Horn et al., 2001; Van Horn and Gazzaniga, 2013). Later came the 1000 Functional Connectomes Project (FCP), which released a curated dataset of 1300 subjects in December 2009^1^. Other recent examples of curating and centralizing multi-site data for open distribution include the Biomedical Informatics Research Network (BIRN), the Functional Biomedical Informatics Research Network (F-BIRN) and The International Neuroimaging Data-sharing Initiative (INDI). INDI hopes to expand on the success of the FCP project by focusing on establishing strong phenotypic datasets to accompany the imaging data^2^.

All of the approaches mentioned thus far have utilized a curation process in which data is manually checked for quality and adherence to project-specific data collection and processing standards. An alternative approach is seen in XNAT Central and the National Database for Autism Research (NDAR), which are centralized repositories for researchers to deposit data^3^ (Hall et al., 2012). Other researchers may then analyze and download the posted neuroimaging datasets. Data that is deposited in these databases is openly available to the community, and therefore must be fully anonymized before upload. XNAT Central does not rely on manual curation to ensure quality and standards, and places the burden of data-verification on the downloader.

Here we propose another approach to providing an Open Neuroscience sharing infrastructure. The proposed approach does not require manual curation by a centralized organization, yet promises stricter adherence to standards than a completely open approach. The key to this approach is a neuroinformatics data management platform called the Collaborative Informatics and Neuroimaging Suite (COINS) (Scott et al., 2011). Researchers have noted the importance of managing data within an informatics platform from the time of collection onward (Mennes et al., 2013). By doing so, data is stored according to generalizable ontologies that can be mapped across studies, sites and even between data management platforms. In addition to offering a framework to organize data for universal mapping, COINS allows researchers to store all of their research data in one place, and then selectively (or globally) share that data in a manner that satisfies the Health Information Privacy and Accountability Act (HIPAA) and secures Protected Health Information (PHI) against accidental exposure. The COINS Data Exchange (DX) is a vehicle for the greater research community to explore, request and download this shared data. The following text outlines the technology used by DX as well as an analysis of the success of the system.

## Materials and methods

The COINS Data Exchange (DX; http://coins.mrn.org/dx) was designed to be a repository where researchers from all over the world can intuitively share data. DX performs two main processes: The first provides an intuitive interface through which researchers can explore, request and download data stored within the COINS database. The second allows researchers who are not already storing their data in COINS to upload that data for sharing. This paper will focus on the first step of the first process: data exploration.

**Figure 1:**
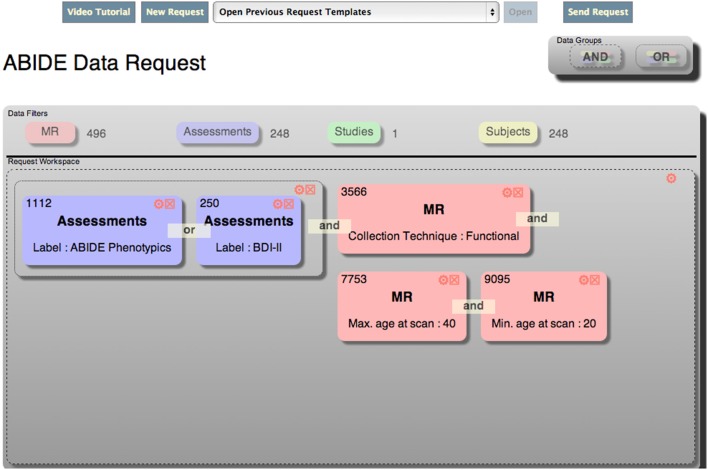
**DX data catalog exploratory filtering tool**.

DX uses a unique exploratory interface to visually construct ad-hoc queries. The interface consists conceptually of a single workspace that represents a *request*. The *request* can have one or more logical *groups*, each of which can have zero or more child *groups* and zero or more *filters*. The *groups* define the logical relationship (and vs. or) between individual children of that group. The workspace is populated with groups and filters by clicking and dragging elements on the screen into the workspace. In addition, filters may be converted to templates by super users, and those templates can be used as a starting point for other users looking for similar data. An example of the interface, which is called the Data Catalog, is shown in Figure 1.

The Data Catalog is made possible by modern web application technologies such as JavaScript and JQuery, HTML5 APIs and CSS3. The development of the Data Catalog was expedited by utilizing Node.js, which facilitated the reuse of libraries in the client and the server (Tilkov and Vinoski, 2010). Historically, web-based applications have been created using one server-side programming language (e.g., java, ruby, php), and a completely different client-side language (JavaScript). Node.js is a paradigm shift from this methodology in that it allows developers to use JavaScript on both the server- and client-side. This code reuse allows developers to program much more efficiently. Other notable companies also using node.js are PayPal, Groupon, eBay, and LinkedIn. PayPal estimates that they were able to create new features twice as fast with fewer people, use 33% fewer lines of code, and generate 40% fewer files when they used node.js as compared to their previous methodology utilizing disparate client and server languages^4^.

### Architecture

**Figure 2:**
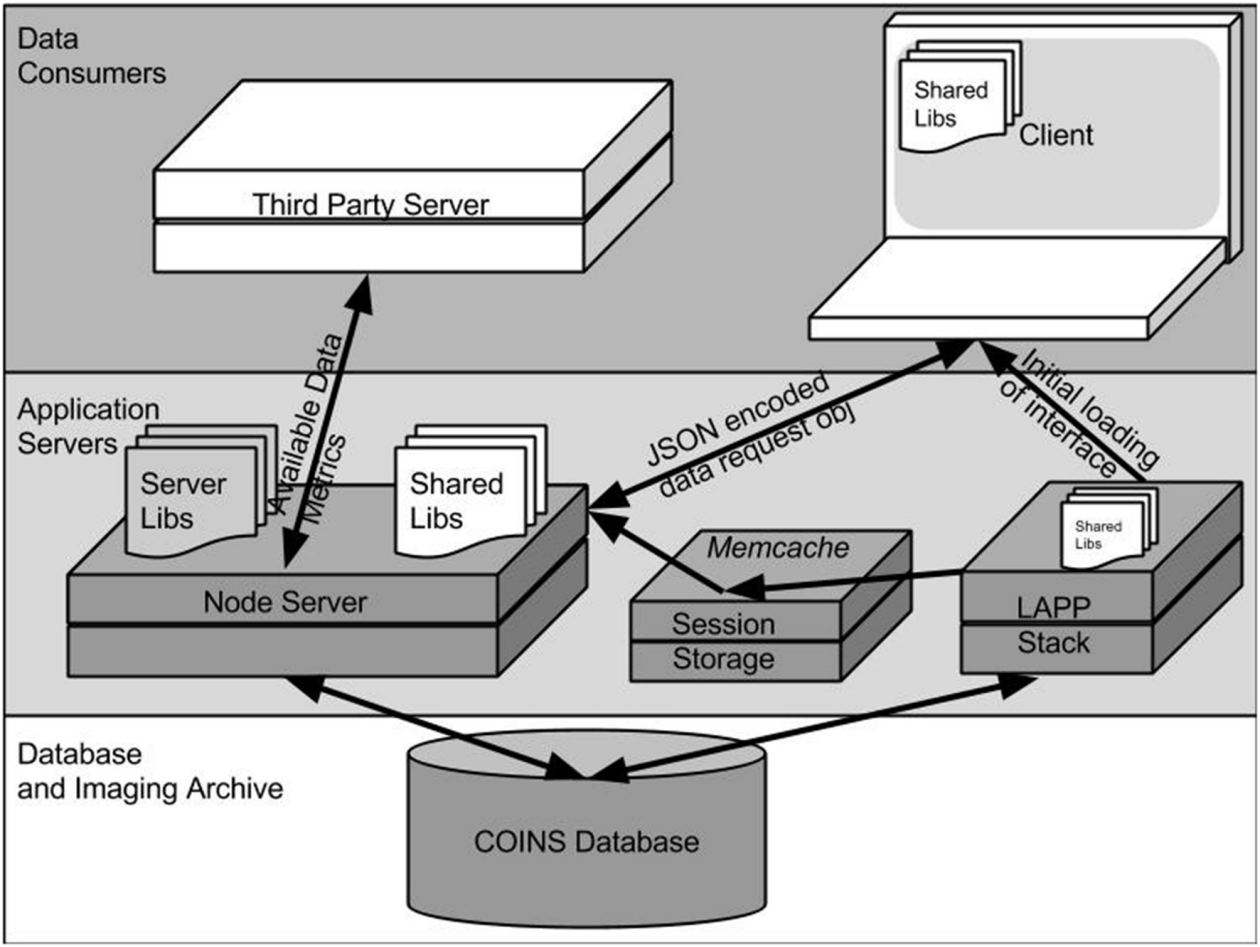
**COINS DX infrastructure**.

The interface is delivered to the browser in the form of a HTML web page dynamically generated by PHP scripts and several javascript libraries. All files are served from the same Linux-Apache-PostgreSQL-PHP (LAPP) servers that host other COINS web applications. Since the Data Catalog is accessed within the COINS web application, security is managed by the COINS Central Authentication System. After a user logs in, their PHP session information is stored on a centralized memcached server, which is accessible to the COINS web application servers as well as the Node.js servers that host the Data Catalog web services (Brad, 2004; Olson et al., 2014). Once the Data Catalog interface has been loaded into the browser, all data queries will be sent to a separate webservice running on a Node.js server. This is illustrated in Figure 2.

The components of the Data Catalog workspace mentioned above, and shown in Figure 1 are represented as JavaScript objects that are defined in the libraries used by both the browser and Node.js server. This allows for objects that represent the user's query (discussed below) to be easily passed from the server to the client and back. This communication is handled via standard asynchronous HTTP(S) requests, and can also be leveraged by other automated services (also shown in Figure 2). For instance, the NIH-Funded SchizConnect Data Federation is working with COINS and XNAT Central and the Human Imaging Database (HID) to create a tool that will automatically compile a comprehensive catalog of data available on both sharing resources. This tool retrieves information about data available in DX via a RESTful API^5^ (SchizConnect Data Federation).

### User interface initialization

When the user interface of the Data Catalog is first initialized, a new Request object is constructed. As part of the construction, the top-group Group object is also constructed, and the filterable modalities are asynchronously retrieved from the server and loaded into the *modalities* property of the new Request object from the server. Each modality represents a mode of data for which there is at least one filterable attribute, and for which statistics should be calculated and displayed.

When the request is modified (either by assigning it a label, or adding a new Filter or Group object, it will be persisted to the server. This is done by calling the Request object's write() method. The write() method utilizes the Request object's toJSON() method, which in-turn calls the toJSON() methods of all child objects (Groups and Filters) in order to properly serialize them. The JSON representation of the object is then sent to the server via a POST HTTP request. On the server, a new Request is once again constructed using the same library that was used on the client. Next, the new Request object's fromJSON() method is invoked, which in-turn recursively calls the fromJSON() methods of each child object to properly unserialize all objects. Following unserialization, the write() method of the server-side Request object is invoked, which overloads the write() method defined in the shared definition of Request. This method writes the relevant properties of the Request to the database, setting the id property to the value assigned by the database. Write() also recursively calls the write() methods of all child objects, so that they are also persisted to the database and assigned identifiers accordingly. Finally, the Request is once-again serialized to JSON, and sent back to the client, where it is unserialized and replaces the current Request object before being rendered.

As filters and groups are added or modified, objects representing those entities are created or updated on the client. Those objects are then encoded into JavaScript Object Notation (JSON) strings and sent to the server for processing via asynchronous HTTP(S) requests (Bray, 2014). The server then parses the JSON strings into proper objects, and forms SQL queries to retrieve statistics about the objects from the COINS database. The resulting statistics are then appended as properties of the objects before JSON encoding them and sending them back to the client where they are used to update the interface with statistics about the current request.

### Javascript data model

**Figure 3:**
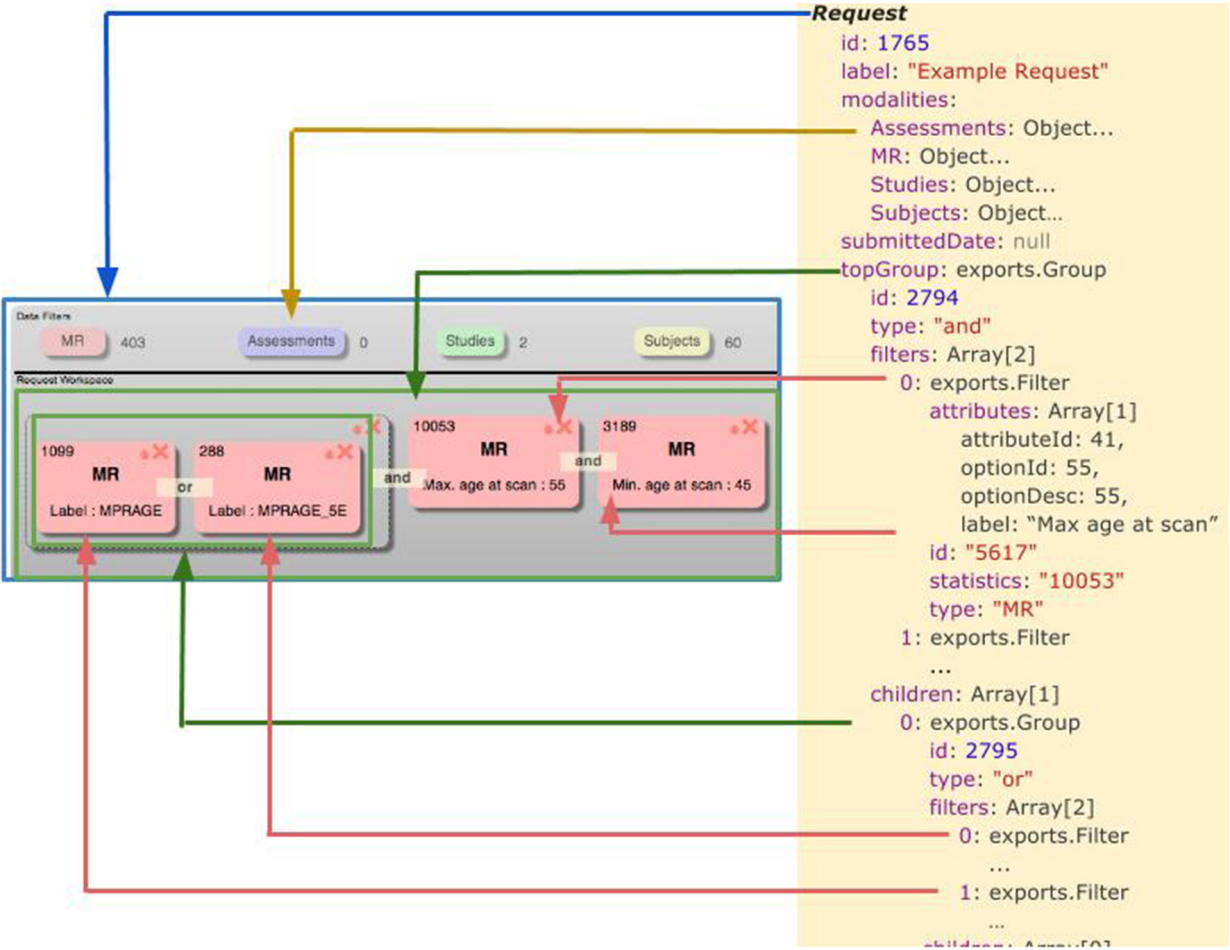
**Simplified model of a data catalog request**.

A simplified model of the Javascript objects that comprise the Data Catalog is shown in Figure 3. Some properties were excluded from the model for clarity. Each object's prototype encapsulates a method to render itself in HTML: a functionality only used on the client. The prototype for each object also contains methods to deconstruct itself into a JSON string that can be sent across a wire. Similarly, each prototype has a method to reconstruct itself from a JSON string or standard object. These methods are employed on both the client and the server to facilitate passing the objects back and forth. Each of the objects illustrated in Figure 3 are explained in more detail below.

The Request object is the top-level object for the Data Catalog UI, and as such, it contains pointers to all other objects relevant to the UI. When a new, blank request is first started by a user, it is assigned a unique identifying integer, which is recorded in the server-side database, and assigned as the id property for the request. Another property of the Request object is populated upon initialization: modalities. Each modality is an object which specifies the type of data for which metrics are to be displayed, and for which filters should be available. A user-specified label may also be associated with the request, and will be persisted to the server-side database as well.

The topGroup property of the Request is populated upon request initialization, and points to a *group* object. Each group object also has a server-defined identifier (id), and properties to list child other groups and filters that reside within the current group. Additionally, groups have a *type* property which can be either “and” or “or.”

Groups may contain zero or more *filter* objects. Filter objects contain a list of attributes, which correspond to rows of the *dx_source_attributes* table mentioned elsewhere in this paper. At present, the user interface only supports one attribute per filter, however, the attributes property is an array in anticipation of future changes. Other properties of each filter object includes the type and statistics associated with the modality of the filter, and a server-issued identifier, which corresponds to a persistent representation of the filter in the COINS database.

Filter *attributes* have properties that define the *source_attribute* to which the property corresponds, as well as the value and its description, as selected by the user for that attribute (*optionId* and *optionDesc*, respectively).

### Server-side processing

Server-side-only libraries extend the object prototypes in order to add database-related functionality. For example, each server-side object prototype (e.g., ServerRequest, ServerGroup, ServerFilter) exposes a method to persist a representation of each object to the database for persistence. Other server-side-only methods generate PL/SQL code to process statistics or metadata about the object in the database. In the case of a filter object, the PL/SQL code inserts the primary keys of all data that matches the filter's *filterAttributes* into a temporary table where it can be intersected or unioned with other filters' data (depending on what type of group the filter is in). As with the JSON (un)serialization methods, all aforementioned methods call their correlate-methods of all child objects (Request.render() will call Group.render() for all Groups in the request, and so on).

### Modalities and filters

**Figure 4:**
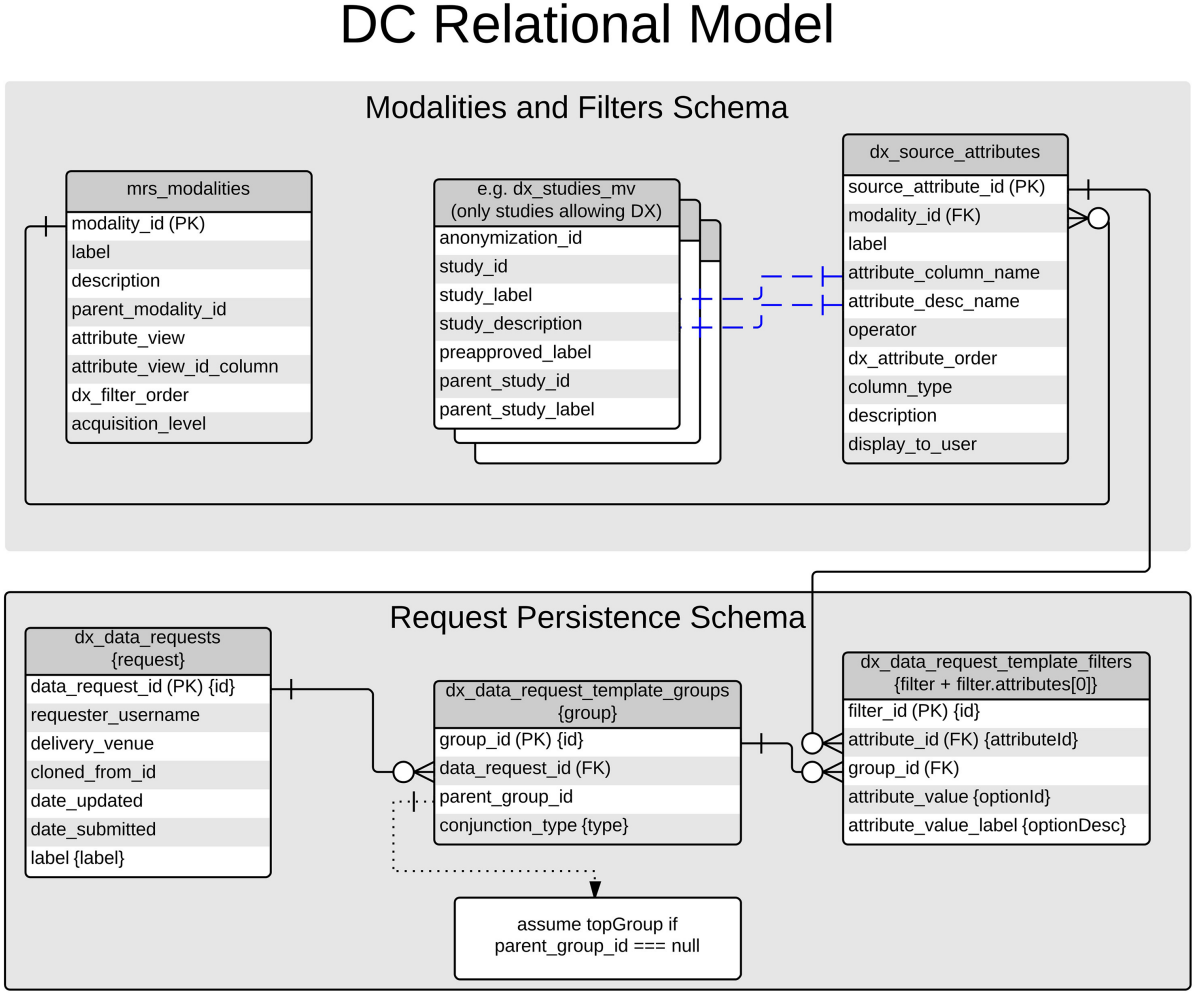
**Database schema for data catalog**.

When a request is first initiated in the client, a list of available modalities and filters is retrieved from the database. These data are manually curated by modifying data stored in the COINS database. Figure 4 depicts the tables discussed in this paper, and a more general understanding of the COINS database was published in 2010 by Scott et al. (2011). The modalities are populated from a table in the database, which consists of modality labels and pointers to the tables and primary keys that they correspond to. Statistics displayed for each modality are calculated by tallying the number of unique primary key values are matched by the user's query. For example, the Study modality corresponds to the a materialized view of available studies and the *anonymization_ids* of subjects that are enrolled in them (*dx_studies_mv*), and the *study_id* primary key.

Filters for each modality are also configured and stored in the COINS database. The table *mrs_source_attributes* stores available attributes which can be filtered upon. Each attribute is linked to a modality via a foreign key constraint. Other columns of mrs_source_attributes specify which columns of the *modality's table* should be used for the available values and value-descriptions available for each filterable attribute. Continuing with the previous example of the Study modality, the value and value-description properties of the *Study Label* filter-attribute correspond to the *label* and *description* columns of *mrs_studies*.

### Query generation

Among the methods exposed by the server-side-only libraries are methods to convert each filter's attributes into SQL queries. Server-side *filter* objects expose a method *generateSQL(), which* generates SQL to select the identifiers of data that is matched by each filter-attribute's “optionId” and “operator.” Similarly, the *group* object exposes a *generateSQL()* method that will *union* (type = *or*) or *intersect* (type = *and*) the modular queries created by the group's filters and child-groups.

In order to allow groups to contain filters of disparate modalities, some additional logic is necessary. SQL modules generated by child-groups and filters of the same modality should be combined using the modality's primary key (e.g., *subjects with age* ≥ *25* AND *subjects with age* ≤ *55*). SQL modules generated by child-groups and filters of varying modalities must be combined using the subject-anonymization-identifier (e.g., *subjects with age* ≥ *25* AND *MR with series label* = “*MPRAGE*”).

This additional logic is assisted by automatically redrawing user-defined groupings every time the user modifies the request object. The re-drawing looks for groups that contain three or more filters, where at least two of which are of the same modality and at least one of which is of a different modality than the others. These filters are then split up into sub-groups according to their modalities: for instance, an “and” group containing two *subject* filters and two *MR* filters will be redrawn to contain two child groups: one for *subject* filters and one for *MR* filters.

The *generateSQL()* methods are called for each object by the object's parent (i.e., The *request* object calls *topGroup.generateSQL()*, which in turn calls the *generateSQL()* method of each of its child groups and filters, and so on). When all method calls have returned, the request receives a single SQL statement that will yield the subject-anonymization-identifiers and modality-specific-primary-keys of all data that is matched by the request. Additionally, the SQL generated by each object can be run independently to retrieve statistics about the amount of data matched by that object.

#### A note about security

Whenever utilizing client-generated values to generate SQL, it is important to screen for SQL injection attacks. The data catalog implements the same security measures practices elsewhere in the COINS application. First, the login-role used by the application does not have read or write access to underlying tables that contain data. Instead, all database reads are performed by selecting data from views, rather than directly from tables. Similarly, all write operations are performed by calls to stored functions. In the case of the data catalog application, the login-role's read access is restricted to data-catalog related views and functions that persist user's requests to the database. Thus, any SQL injection attacks would not reveal any more information than is readily available through the user interface. For the sake of added caution, other standard protections are also implemented, such as type-checking, query parameter binding, user authentication, a 30-min logout window, and record-modification history logging.

## Results

### Application and features

The Data Catalog is a critical component of the COINS Data Exchange. It allows users to construct complex ad-hoc queries against sharable data in the COINS database in an exploratory way to form a request for data. After constructing a request, the request can be submitted, which will notify all COINS users that own the data being requested that some of their data is being requested. The submitted request can be accepted or denied by the data owners after the requester and owner have exchanged messages through the integrated messaging system. All messages are stored indefinitely, and can be used as official documents or an audit trail if needed. If one data-owner approves the request, and another denies it, only the approved subset of data will be made available to the requester. Data associated with accepted requests is packaged and zipped on the COINS servers, and the requester is notified when the package(s) are ready for download. The packaging, zipping, and download process is also quite interesting, but will not be explained in detail here.

### Integration with COINS

Data collected via COINS is easily shareable in DX. Study administrators are provided very fine-grained control over which data is shared: individual subject types, subjects, scans, instruments or assessments may be excluded or included. Additionally, sharing benefits from the centralized approach of COINS. Studies that have collected data using shared instruments can now expose their data to sharing more easily. This allows a Data Catalog user to request data from two studies that have collected data using the Balanced Depression Inventory II (BDI-II) with a single filter (*Instrument label* = “BDI-II”).

### Data shared

The first publicly-available dataset to be shared on COINS DX was the^6^ ABIDE dataset. Released in the COINS DX on August 30th 2012, the ABIDE dataset consists of functional and structural imaging and phenotypic data from more than 1000 participants gathered from 15 different sites around the world (Nooner et al., 2012). The ABIDE dataset was imported into the COINS data management system and made available for sharing. To date, 166 individuals have downloaded over 2450 GB of ABIDE data through COINS DX.

Next, the NKI Rockland Sample made their first data release in March 2013^7^. Unlike the ABIDE dataset, the NKI Rockland Sample dataset was collected directly using COINS. This allows the NKI research team to make periodic releases by simply selecting which subjects and data is ready to be shared in DX. Their changes are reflected instantly in the Data Catalog. Researchers requesting access to the NKI Rockland Sample dataset require individual approval after agreeing to a DUA. Despite the more rigorous approval process, over 1200 GB of data have been approved for sharing and downloaded by 15 researchers from around the world.

## Discussion

The COINS Data Catalog harnesses modern web technologies to extend a popular neuroinformatics platform for use in the context of Open Neuroscience. The architecture of the application has proven flexible, maintainable, and secure. Moreover, two large datasets have been successfully shared on an international scale. One of those datasets was collected and compiled outside of COINS, then successfully imported. The other dataset is part of an ongoing collection effort using COINS tools, and can be easily curated by the data owners. Over all, over 3500 GB of data have been shared through the COINS Data Exchange since September 2012.

There remains a huge potential to share an increasing amount of data using DX: There are currently over 500 studies being managed with COINS. These studies have collected 342,000 clinical assessments and 31,400 MRI and MEG scan sessions from 22,100 participants at many sites across the United States including The Mind Research Network, Nathan Kline Institute, University of Colorado—Boulder, Olin Neuropsychiatry Research Center (King et al., 2014). Each of these studies can easily elect to allow some or all of their data to be explored and requested through DX.

As more studies elect to share their data through DX, the greater the number of filtering options will become during data exploration. If the number of filtering options grows too large, it may become difficult for a researcher to locate the options that apply to their own interests. It is important therefore to create data dictionaries and ontological mappings for the large amount of data currently stored within COINS. Such mappings will allow for multi-level filtering options that correspond to other popular common data elements.

Looking ahead, the developers of the COINS DX are excited to implement more features to aid sharing within the Open Neuroscience community. Dynamic requests are being developed, which will periodically alert researchers if new data is made available which matches one of their existing filters. Further improved API performance and documentation is on the way, and will aid in integration with projects such as SchizConnect (SchizConnect Data Federation) and Neurodebian^8^.

### Conflict of interest statement

The authors declare that the research was conducted in the absence of any commercial or financial relationships that could be construed as a potential conflict of interest.

## Footnotes

^1^https://fcon_1000.projects.nitrc.org/ accessed 1/28/2014

^2^http://fcon_1000.projects.nitrc.org/indi/docs/INDI_MISSION_STATEMENT.pdf

^3^ XNAT Central https://central.xnat.org/

^4^https://www.paypal-engineering.com/2013/11/22/node-js-at-paypal/ Dec 23, 2013

^5^http://niacal.northwestern.edu/projects/18

^6^http://fcon_1000.projects.nitrc.org/indi/abide/

^7^http://neuro.debian.net/

^8^http://neuro.debian.net/
