# Corrigendum: Harnessing modern web application technology to create intuitive and efficient data visualization and sharing tools

**DOI:** 10.3389/fninf.2015.00014

**Published:** 2015-06-05

**Authors:** Dylan Wood

**Affiliations:** The Mind Research NetworkAlbuquerque, NM, USA

**Keywords:** neuroinformatics, NeuroDebian, COINS, data sharing, open neuroscience

**Footnotes 6 and 7:**

Should both refer to the ABIDE dataset: http://fcon_1000.projects.nitrc.org/indi/abide/

**Footnote 8:**

Should reference the article below, in addition to the NeuroDebian website: http://neuro.debian.net.

Halchenko, Y. O., and Hanke, M. (2012). Open is not enough. Let's take the next step: an integrated, community-driven computing platform for neuroscience. *Front. Neuroinform*. **6**:22. doi: 10.3389/fninf.2012.00022. http://journal.frontiersin.org/article/10.3389/fninf.2012.00022/pdf

## Conflict of interest statement

The author declares that the research was conducted in the absence of any commercial or financial relationships that could be construed as a potential conflict of interest.

